# The Lack of Natural IgM Increases Susceptibility and Impairs Anti-Vi Polysaccharide IgG Responses in a Mouse Model of Typhoid

**DOI:** 10.4049/immunohorizons.2200088

**Published:** 2022-12-01

**Authors:** Akhil S. Alugupalli, Matthew P. Cravens, Justin A. Walker, Dania Gulandijany, Gregory S. Dickinson, Gudrun F. Debes, Dieter M. Schifferli, Andreas J. Bäumler, Kishore R. Alugupalli

**Affiliations:** * Department of Microbiology and Immunology, Sidney Kimmel Medical College, Thomas Jefferson University, Philadelphia, PA; † Department of Microbiology, University of Pennsylvania School of Veterinary Medicine, Philadelphia, PA; ‡ Department of Medical Microbiology and Immunology, School of Medicine, University of California at Davis, Davis, CA; § Sidney Kimmel Cancer Center, Thomas Jefferson University, Philadelphia, PA

## Abstract

Circulating IgM present in the body prior to any apparent Ag exposure is referred to as natural IgM. Natural IgM provides protective immunity against a variety of pathogens. *Salmonella enterica* serovar Typhi (*S.* Typhi) is the causative agent of typhoid fever in humans. Because mice are not permissive to *S.* Typhi infection, we employed a murine model of typhoid using *S. enterica* serovar Typhimurium expressing the Vi polysaccharide (ViPS) of *S.* Typhi (*S.* Typhimurium strain Typhi RC60) to evaluate the role of natural IgM in pathogenesis. We found that natural mouse IgM binds to *S.* and *S.* Typhimurium. The severity of *S.* Typhimurium infection in mice is dependent on presence of the natural resistance-associated macrophage protein 1 (*Nramp1*) allele; therefore, we infected mice deficient in secreted form of IgM (sIgM) on either a *Nramp1*-resistant (129S) or -susceptible (C57BL/6J) background. We found that the lack of natural IgM results in a significantly increased susceptibility and an exaggerated liver pathology regardless of the route of infection or the *Nramp1* allele. Reconstitution of sIgM^−/−^ mice with normal mouse serum or purified polyclonal IgM restored the resistance to that of sIgM^+/+^ mice. Furthermore, immunization of sIgM^−/−^ mice with heat-killed *S.* Typhi induced a significantly reduced anti-ViPS IgG and complement-dependent bactericidal activity against *S.* Typhi in vitro, compared with that of sIgM^+/+^ mice. These findings indicate that natural IgM is an important factor in reducing the typhoid severity and inducing an optimal anti-ViPS IgG response to vaccination.

## INTRODUCTION

*Salmonella enterica* serovar Typhi (*S.* Typhi) is the causative agent of typhoid fever in humans. Global estimates reported by the Centers for Disease Control and Prevention indicate that 21.6 million cases of typhoid fever occur each year resulting in 226,000 deaths (1). The rapid emergence of multiple drug-resistant strains of *S.* Typhi now complicates the treatment of typhoid (2). Typhoid is a vaccine-preventable disease, and vaccination of high-risk populations such as infants and young children is considered the most promising strategy for control (3). Vi polysaccharide (ViPS) is a target for immune responses, and anti-ViPS Abs correlate with protection against typhoid fever (3–5). Three types of vaccines are currently available (5): 1) a live attenuated vaccine (Vivotif), 2) a subunit vaccine composed of plain ViPS (Typhim Vi or Typherix) (5, 6), and 3) ViPS conjugated to a variety of carrier proteins such as a recombinant exoprotein A from *P. aeruginosa* (7), CRM197, a nontoxic mutant of diphtheria toxin (8), tetanus toxoid (9), or diphtheria toxoid (10). The efficacy of live-attenuated oral vaccine is ~60% (6, 7), and the plain ViPS, an injectable vaccine, is ~55% in older children and adults (5, 11). Importantly, plain ViPS vaccines do not induce optimal Ab responses in children <2 y of age. ViPS conjugated to recombinant exoprotein A, as well as tetanus toxoid, can induce anti-ViPS responses in infants and young children with 80–90% efficacy (7, 12, 13). ViPS conjugated to CRM197 was not very immunogenic in multinational clinical trials in the Philippines, Pakistan, and India (8, 14). Surprisingly, the secondary and tertiary boosters of this ViPS conjugate vaccine in these typhoid-endemic countries did not yield any increase in the IgG titers to ViPS (8, 14). The reasons for this lack of an optimal anti-ViPS IgG response are not understood.

One of the barriers to advancing the treatment and prevention of typhoid is the lack of a suitable animal model to study *S.* Typhi infection (6). Because *S*. Typhi is strictly human adapted, *S. enterica* serovar Typhimurium (*S.* Typhimurium), a natural pathogen of mice causing a “typhoid-like” systemic disease in mice, became the most widely used bacterium to understand pathogenesis and immunity in inbred mice (e.g., C57BL6/J and 129Sv strains). Organs of the mononuclear phagocytic system, such as liver and spleen, are the major sites of replication of *S.* Typhimurium during infection in mice (15, 16). The relative susceptibility or resistance to *S.* Typhimurium is dependent on a single nucleotide polymorphism in the *Slc11a1* gene that encodes an ion transporter commonly referred to as natural resistance-associated macrophage protein 1 (Nramp1) (17). Humans and 129S mice have a functional *Nramp1* allele, whereas C57BL/6 mice have the *Nramp1* mutant allele that encodes for a nonfunctional protein. Consequently, C57BL/6J mice are more susceptible to *S.* Typhimurium infection than is the 129S strain (15). *S.* Typhimurium shares 90% of its genes with *S.* Typhi (18). However, ~600 *S.* Typhi genes, including those encoding for the ViPS biogenesis, a well-known virulence factor, are absent in *S.* Typhimurium, and several genes found in *S.* Typhimurium are pseudogenes in *S.* Typhi (18, 19). To understand the function of ViPS in vivo, Bäumler and colleagues (20) introduced all of the *S.* Typhi genes required for ViPS biogenesis in *S.* Typhimurium. Using this “chimeric” *S.* Typhimurium strain TH170 (20), roles for ViPS in resisting C3 deposition and complement receptor 3–mediated clearance (21), evading host TLR4 recognition (22, 23), and microbe-guided neutrophil chemotaxis (24) were identified. The length of LPS of *S.* Typhimurium is significantly elongated compared with *S*. Typhi. In *S.* Typhimurium the length of LPS is controlled by *FepE* gene product, which is a pseudogene in *S.* Typhi (19). To modify the surface characteristics of *S.* Typhimurium to resemble those of *S.* Typhi, the *S.* Typhimurium strain TH170 was further engineered by deleting the *FepE* gene (25). This strain of *S.* Typhimurium, referred to as RC60, exhibits cell surface characteristics of *S.* Typhi and captures certain aspects of *S.* Typhi pathology in mice (25). Using the *S.* Typhimurium strain RC60, we were able to identify several features of the anti-ViPS Ab repertoire required for protective immunity in vivo (16, 26, 27).

Circulating IgM present in the body prior to any apparent Ag exposure is referred to as natural IgM (28–30). Natural IgM in mice is produced mainly by B1a cells and marginal zone B (MZB) cells, can bind to evolutionarily conserved Ags on pathogens, and plays an important role in protective immunity (28–33). In contrast, Ab responses to a variety of bacterial Ags, including ViPS, are generated from B1b cells (34–38). Mice deficient in activation-induced cytidine deaminase (AID) have Abs that can neither undergo Ig isotype switching nor somatic hypermutation of the variable regions (39). The absence of functional AID in humans leads to significantly increased basal serum IgM levels, also known as hyper-IgM syndrome type 2 (40). Similarly, AID^−/−^ mice have a 2- to 3-fold increase in basal levels of natural IgM (39). Using AID^−/−^ mice, we have shown that unmutated IgM Abs against ViPS can confer protective immunity against *S.* Typhimurium RC60 infection (16). A striking observation was that the necrotic regions of the liver of unimmunized AID^−/−^ mice infected with *S.* Typhimurium RC60 were significantly smaller than those in unimmunized wildtype mice (16). We hypothesized that this decrease in pathology is attributable to having 2- to 3-fold higher levels of natural IgM in the AID^−/−^ mice than in naive wild-type mice (39). This finding is consistent with the fact that passive immunization of mice with high doses of polyclonal human IgM can reduce *S.* Typhimurium burden in various tissues (41). In the current study, we determined the role of natural IgM in typhoid susceptibility in a murine model by infecting mice deficient in the secreted form of IgM (sIgM^−/−^) with *S.* Typhimurium RC60 and the impact of natural IgM on the anti-ViPS IgG response to immunization.

## MATERIALS AND METHODS

### Mice

The Thomas Jefferson University Institutional Animal Care and Use Committee has approved these studies. Mice were housed in microisolator cages with free access to food and water and were maintained in a specific pathogen-free facility. Mice that lack sIgM but have the membrane-bound form of IgM (sIgM^−/−^) have been previously described (42). The sIgM^−/−^ mice used are on a 129Sv (129Sv.sIgM^−/−^) or C57BL/6J (B6.sIgM^−/−^) background. Control wild-type (129S1/SvImJ; stock no. 002448) and (C57BL/6J; stock no. 000664) mice were purchased from The Jackson Laboratory (Bar Harbor, ME). The sIgM genotype/phenotype is confirmed either by PCR or IgM ELISA (42). To determine the variation in the *Nramp1* allele in the 129Sv.sIgM^−/−^ and B6.sIgM^−/−^ mice, a 514-bp fragment of the Nramp1 gene was amplified by PCR using primers 5′-AAGTGACATCTCGCCATAGGTGCC-3′ and 5′-TTCTCTCACCATAGTTATCCAAG AAG-3′ (forward and reverse, respectively). The purified PCR product was then sequenced using the primer 5′-CCCCCATCTATGTTATCACCC-3′ (43). Sequencing for the point mutation that results in a glycine to aspartate coding change at position 169 within *Nramp1* was as described (44). Age-matched (8- to 12-wk-old) mice of both sexes were used for all experiments.

### Infections

Mice were infected with a chimeric strain of *S.* Typhimurium (strain RC60) that expresses the *S*. Typhi genes necessary for ViPS synthesis, export, and regulation as in *S*. Typhi (25). Strain RC60 was grown to an OD_600_ of ~1.0 in Luria–Bertani (LB) broth with 10 mM NaCl. The expression of ViPS was assessed by a slide agglutination test using a commercial Vi mAb reagent (Statens Serum Institute Diagnostica, Copenhagen, Denmark; lot 188L-8). Bacteria were washed twice in Dulbecco’s PBS (DPBS), and 100 μl of DPBS containing 3 × 10^4^ CFU was injected i.p. or i.v. At 3 d postinfection liver and spleen were collected, and the tissues were processed using a Minilys tissue homogenizer (Bertin Technologies, Montigny-le-Bretonneux, France). The bacterial burdens in the liver and spleen homogenates, as well as blood collected into anticoagulant, were determined by plating serial 10-fold dilutions on LB agar plates followed by counting CFU (16, 26, 27).

### Histopathology analysis

Liver tissues obtained on day 3 postinfection were fixed in 10% buffered formalin, and 4 μM paraffin-embedded sections were stained with H&E. The specimen slides were scanned at ×20 magnification on an Aperio CS2 ScanScope (Leica Biosystems), and total, necrotic, and infiltration areas composed of lymphocytes and other mononuclear cells in the entire specimen were quantified using Aperio ImageScope software (Leica Biosystems) (16, 26).

### Reconstitution of mice with naive mouse serum or IgM

Mice sufficient or deficient in IgM (i.e., 129Sv.sIgM^+/+^ or 129Sv.sIgM^−/−^) were injected i.p. with 150 μl of sterile-filtered serum obtained from naive 129S1/SvImJ, 129Sv.sIgM^−/−^ mice or 200 μl of PBS containing 200 μg of purified polyclonal IgM purchased from Rockland Immunochemicals (Pottstown, PA). The commercial IgM preparation was purified from normal serum and contains 0.1% azide and 0.5 M NaCl and 0.1 M Tris (item no. 010–0107; Rockland Immunochemicals, Pottstown, PA). Therefore, the IgM preparation was dialyzed against PBS (1000 ml) twice in a 10,000 Da molecular mass cutoff Slide-ALyzer MINI dialysis device (Thermo Fisher Scientific). Because IgM has the shortest half-life among all of the Ab isotypes (45), blood was sampled a day after transfer to confirm IgM levels by ELISA and at the same time the mice were challenged with strain RC60 i.p.

### Depletion of neutrophils

One day before infection, mice were injected i.p. with 300 μg of anti-mouse Ly6G/Ly6C (Gr-1) mAbs in 200 μl of PBS (clone RB6-8C5; Bio X Cell, Lebanon, NH) or PBS alone. As previously reported, this treatment results in neutrophil depletion for 4 d (46).

### Immunization

For whole bacterial immunization, mice were injected i.p. with 3 × 10^8^ CFU of heat-killed *S.* Typhi strain Ty2 (grown in LB broth containing 10 mM NaCl) in 100 μl of DPBS (16). Blood samples were obtained 0, 7, 14, 21, or 28 d following immunization and stored at −20°C.

### ELISA

ViPS-specific IgM and IgG in the blood were measured by coating 96-well microtiter plates (Nunc MaxiSorp; Invitrogen, Carlsbad, CA) with 2 μg/ml ViPS purified from *S.* Typhi clinical isolate C6524 (47) in DPBS overnight at room temperature. All plates were washed and blocked with 1% BSA in PBS (pH 7.2) (blocking buffer) for 1 h at room temperature. Blood samples from immunized mice were diluted to 1:25 for IgG detection and 1:50 for IgM detection in blocking buffer, and samples were centrifuged (800 × *g* for 10 min) and cell-free supernatant was used. The dilutions, 1:25 for IgG and 1:50 for IgM, were chosen after evaluating various serum dilutions within the linear range by ELISA. Bound IgM or IgG was measured using HRP-conjugated goat anti-mouse IgM or IgG (Bethyl Laboratories, Montgomery, TX). Because ViPS-specific mouse IgM and IgG reference standards are not available, and the ViPS-specific Abs in mice are likely to be of oligoclonal nature with varying affinities, the Ag-specific Ab levels in the current study were interpreted as nanogram per microliter “equivalents” using normal mouse serum IgM or IgG standards (Bethyl Laboratories, Montgomery, TX).

### Serum bactericidal assay

A serum bactericidal assay was performed as previously described (48). In brief, log-phase cultures (OD_600_ of 0.5 at 37°C) of *S.* Typhi strain Ty2 were prepared in LB broth with 10 mM NaCl. Bacterial cells were washed in DPBS, and the bacterial cell density was adjusted to 2.5–5.0 × 10^4^ CFU/ml in DPBS. The expression of ViPS was assessed by a slide agglutination test using a commercial Vi mAb reagent (Statens Serum Institute Diagnostica, Copenhagen, Denmark; lot 188L-8). Serum samples were heat-inactivated by incubating at 56°C for 30 min prior to use in the assay. Ten microliters of *S*. Typhi strain Ty2 in DPBS (250–500 CFU) was added to each well of a round-bottom polypropylene 96-well plate containing 50 μl of heat-inactivated serum in serial dilutions, 12.5 μl of baby rabbit complement (Pel-Freez Biologicals, Rogers, AR), and 27.5 μl of DPBS. Triplicate samples of each dilution were incubated for 120 min at 37°C with gentle rocking, and 10 μl of this mixture were plated on LB agar plates for counting CFU. Serum bactericidal Ab titers are defined as the reciprocal of the highest dilution that produced 50% killing in relationship to control wells containing complement, but no mouse serum. Naive mouse serum served as a negative control, and serum from either mice immunized with heat-killed *Escherichia coli* strain W3110 expressing pDC5 plasmid, which contains the genes necessary for the synthesis and export of ViPS (49), or *S*. Typhi anti-Vi human IgG standard (lot R1, 2011; U.S. Food and Drug Administration, Silver Spring, MD) served as two independent positive controls.

### Natural IgM binding assay

*S.* Typhimurium strains IR715 and RC60 and *S.* Typhi strain Ty2 were grown in LB broth. Overnight bacterial cultures were washed twice in PBS, then resuspended in 25% normal mouse serum (from C57BL/6 mice) in PBS to achieve a final concentration 1 × 10^9^ CFU/ml. After a 1-h incubation at room temperature, the bacterial cells were washed three times in PBS by centrifugation (16,000 × *g* for 8 min). Bound serum proteins were eluted by resuspending the bacterial pellet in 100 μl of 0.1 M glycine-HCl for 30 min. Following centrifugation (16,000 × *g* for 8 min), 80-μl supernatants were collected and neutralized with 20 μl of 1 M Tris-HCl buffer (pH 8.0). Two microliters of these samples was mixed with 48 μl of Laemmli buffer containing 2-ME and boiled for 10 min. Five microliters of the samples was subjected to SDS-PAGE and Western blot analyses. After electrotransfer, the polyvinylidene difluoride membranes (Merck Millipore) were blocked for 1 h in 2% BSA in PBS containing 0.25% Tween 20. The blots were probed either with HRP-conjugated goat anti-mouse IgM Ab or with HRP-conjugated goat anti-mouse IgG Ab (Bethyl Laboratories, Montgomery, TX). After a 1-h incubation, the blots were washed with PBS containing 0.25% Tween 20, developed using Super-Signal West Dura substrate (Thermo Scientific, Rockford, IL), and images were obtained on an imager, Protein Simple FluorChemR (Biotechne, Minneapolis, MN).

### Statistical analysis

Data presented throughout depict pooled data from at least two independent experiments. Statistics were performed using the Prism 5 software program (GraphPad Software, La Jolla, CA), and the statistical tests are indicated in the figure legends.

## RESULTS

### *Normal serum IgM binds efficiently to* S. *Typhi and* S. *Typhimurium*

**FIGURE 1.**
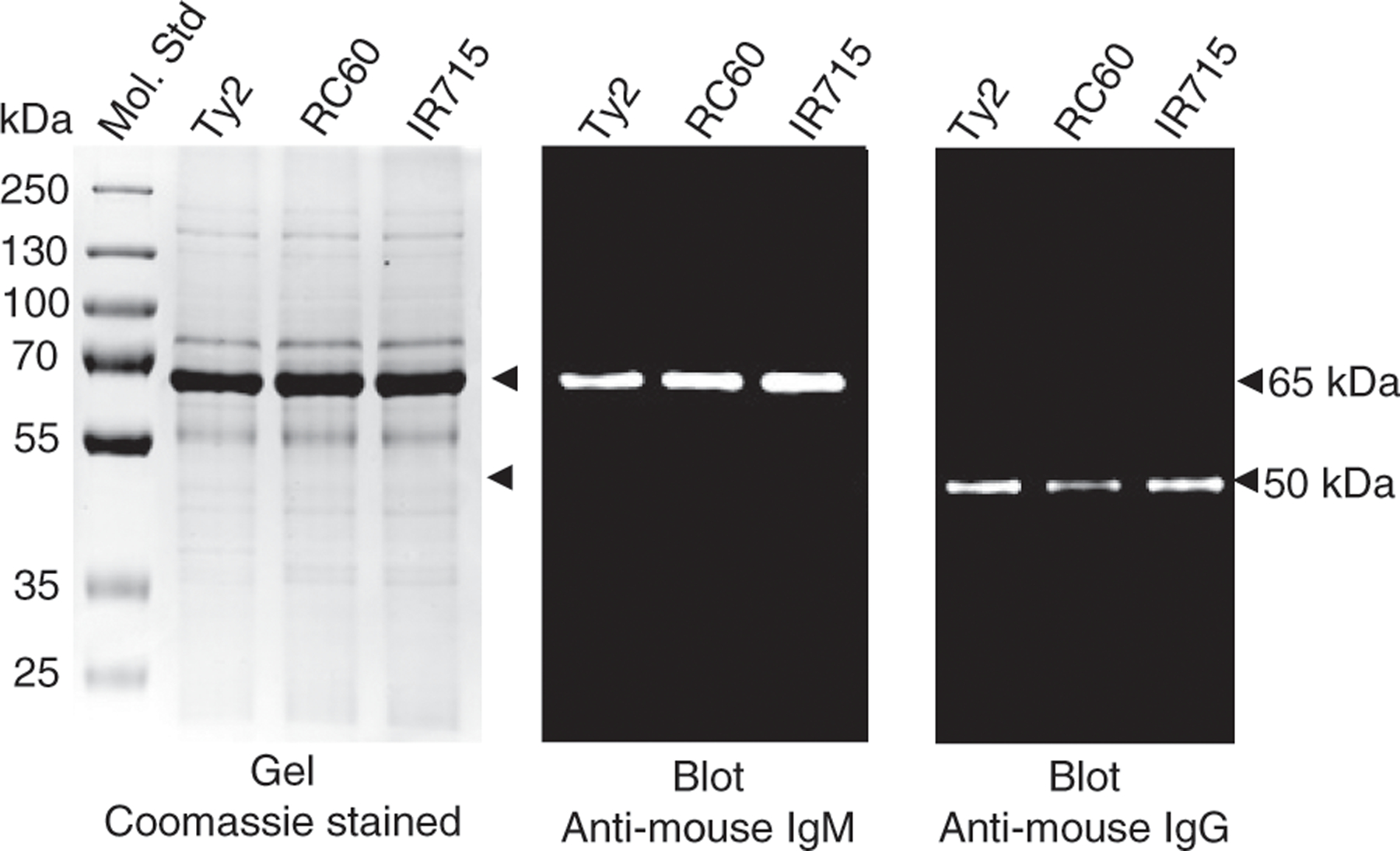
Normal serum IgM binds to S. Typhi and S. Typhimurium. Normal mouse serum from C57BL/6 mice was incubated with indicated bacterial strains. Serum proteins bound to bacteria were eluted and subjected to SDS-PAGE and Western blot analyses. The gel was stained with Coomassie Blue, and the blots were developed using HRP-conjugated anti-mouse IgM or anti-mouse IgG. Arrowheads at 65 and 50 kDa correspond to IgM and IgG H chains under reducing conditions.

Natural IgM binds to evolutionarily conserved Ags of bacteria and other microorganisms (31, 32). This property of natural IgM plays an important role in the host defense. To test whether natural IgM binds to *S.* Typhi and *S.* Typhimurium, we performed a mouse IgM binding assay using serum obtained from naive C57BL6/J mice. This assay is identical to the one developed for human IgM binding to *Salmonella*, except we used mouse serum instead of human serum (50). We found that normal mouse serum IgM adsorbed more efficiently than any other serum proteins to *S.* Typhi and *S.* Typhimurium, as evidenced by the appearance of a major band at the 65 kDa position in the Coomassie gel, which corresponds to the molecular mass of the IgM H chain under reducing conditions (Fig. 1). Immunoblot analysis indeed confirmed that this band is reactive to anti-mouse IgM (Fig. 1). To test whether natural mouse IgG also binds to *Salmonella*, we incubated an identical blot with goat-anti mouse IgG Abs. Although the band is very faint in the Coomassie gel, we observed a 50-kDa band with molecular mass of the IgG H chain under reducing conditions, reactive with anti-mouse IgG (Fig. 1). A comparable reactivity to *S.* Typhimurium strain IR715, which does not express ViPS, indicates that natural IgM and IgG bind to the surface of these bacteria, and to Ags other than ViPS Ag (Fig. 1). These data demonstrate that natural IgM and to a small extent natural IgG bind to *S.* Typhi and *S.* Typhimurium.

### Mice deficient in sIgM are hypersusceptible to typhoid

**FIGURE 2.**
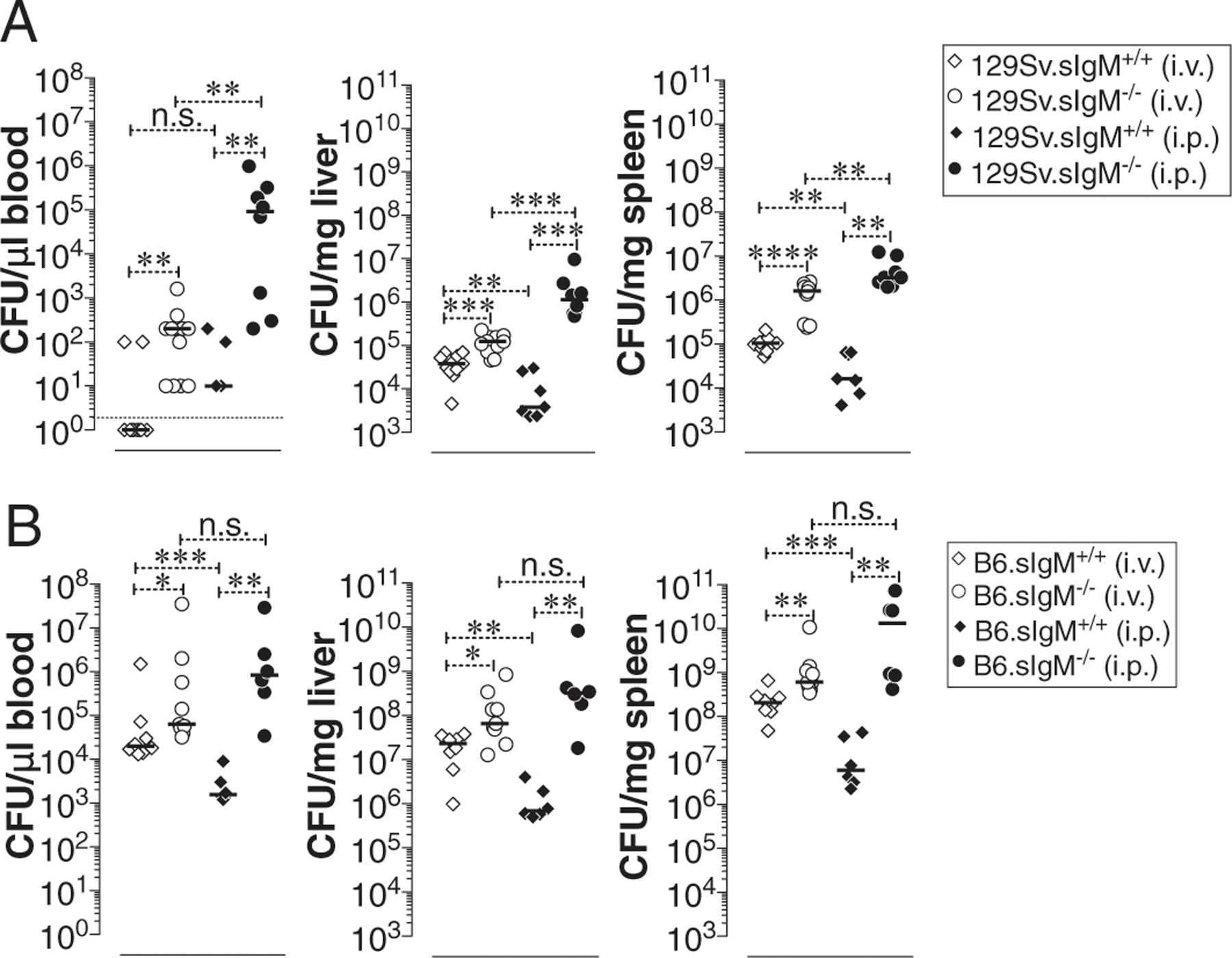
Increased bacterial burden in mice deficient in preimmune or natural IgM Abs. (**A** and **B**) Mice deficient in sIgM on 129S (*Nramp1* r allele) (A) or on C57BL/6J (B6; *Nramp1* s allele) (B) background were infected with 3 × 10^4^ CFU of ViPS expressing *S*. Typhimurium strain RC60 either i.v. or i.p. At 3 d postinfection mice were sacrificed and bacterial burden in the liver and spleen was determined by plating serial 10-fold dilutions of tissue homogenates followed by colony counting. Each dot represents an individual mouse, and the black bar represents the mean. Dotted line in (A) indicates the limit of detection in blood. The data represent a pool of two independent experiments. Statistical differences were determined by a Mann–Whitney *U* test. *p < 0.05, **p < 0.01, ***p < 0.001. n.s., not significant.

To test whether natural IgM contributes to the control of *S.* Typhimurium in vivo, we infected wild-type 129S1/SvImJ mice (129S.sIgM^+/+^) or mice deficient in sIgM (129S.sIgM^−/−^) with *S.* Typhimurium strain RC60 either i.v. or i.p. Compared with 129S.sIgM^+/+^ mice, the 129S.sIgM^−/−^ mice had a significantly increased bacterial burden in the blood, liver, and spleen regardless of the route of infection (Fig. 2A). *S.* Typhimurium susceptibility in mice is determined by the type of *Nramp1* allele (17, 51–53). 129S1/SvImJ mice have the *Nramp1*-resistant alleles as in humans, and *S.* Typhimurium infection causes a chronic infection (15, 54). Conversely, the C57BL/6J mouse strain has the *Nramp1*-susceptible alleles, and *S.* Typhimurium infection causes an acute and lethal infection (15, 54). To test whether the *Nramp1*-resistant or -susceptible allele has an impact on the role of natural IgM-mediated protective immunity, we also infected C57BL/6J mice sufficient or deficient in sIgM (B6.sIgM^+/+^ or B6.sIgM^−/−^) with *S.* Typhimurium strain RC60. As previously shown for the *S.* Typhimurium strain SL1344 (15), C57BL/6J mice are more susceptible than 129S1/SvImJ mice to *S.* Typhimurium strain RC60 infection, indicated by 100- to 1000-fold higher bacterial burden (Fig. 2). Importantly, B6.sIgM^−/−^ mice had a significantly increased susceptibility compared with the B6.sIgM^+/+^ mice (Fig. 2B). These data indicate that natural IgM contributes to the control of murine typhoid.

### Mice deficient in sIgM exhibit increased liver pathology

**FIGURE 3.**
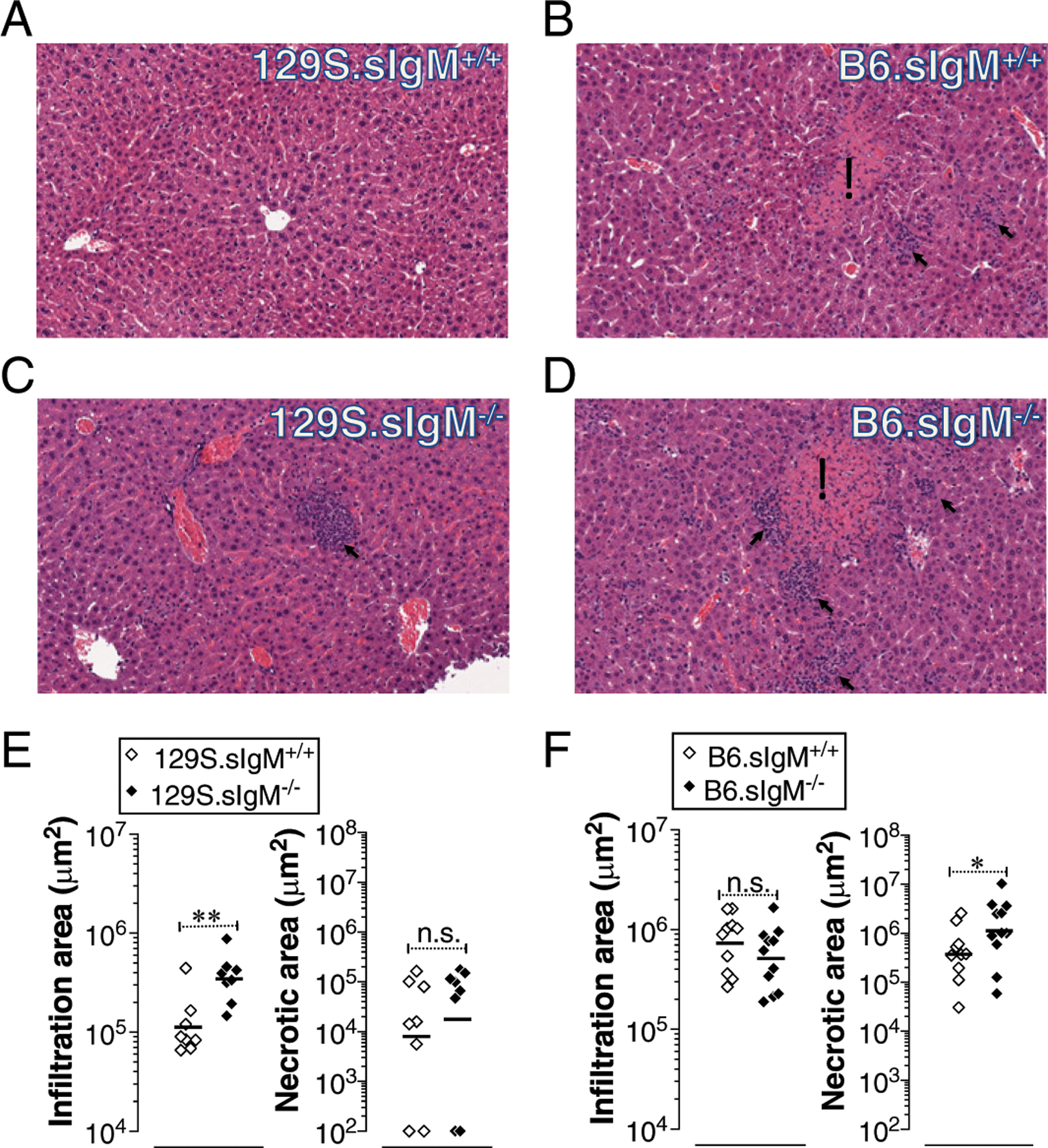
Increased liver pathology in mice deficient in sIgM. (**A**–**F**) Mice deficient in sIgM either on 129S (*Nramp1* r allele) (A, C, and E) or on C57BL/6 (B6; *Nramp1* s allele) (B, D, and F) background were infected with 3 × 10^4^ CFU of ViPS expressing *S*. Typhimurium strain RC60 i.p. and H&E-stained liver sections of 3-d postinfected mice were analyzed. In (A)–(D), necrotic lesions are indicated with an asterisk and mononuclear cellular infiltration areas are indicated with arrows (original magnification ×20). (E and F) Quantification of liver pathology. Each dot represents an individual mouse, and the bar represents the mean. The data represent a pool of two independent experiments. Statistical differences were determined by a Mann–Whitney *U* test. *p < 0.05, **p < 0.01. n.s., not significant.

The murine typhoid-induced liver lesions consist of areas of mononuclear cell infiltrates and/or necrosis (16, 26) akin to the lesions described in the liver biopsies of human typhoid patients (55–57). Compared to the i.v. route of infection, the i.p. route of systemic infection resulted in higher bacterial burden in the absence of sIgM in both B6 and 129S backgrounds (Fig. 2). To test whether the increased susceptibility also reflects an exacerbated liver pathology, H&E-stained tissues specimens were evaluated by an observer-blind histopathological analysis. As expected, the relatively resistant 129S.sIgM^+/+^ mice showed smaller areas of mononuclear cell infiltration that increased significantly in 129S.sIgM^−/−^ mice (Fig. 3A versus Fig. 3B) without a significant difference in the size of necrotic areas (Fig. 3E). In contrast, the necrotic areas in the livers of B6.sIgM^−/−^ mice were significantly larger compared with B6.sIgM^+/+^ mice, whereas the areas of mononuclear cell infiltration were similar (Fig. 3B, 3D, 3F). These data indicate that natural IgM is a factor controlling the extent of liver pathology during the early stages of typhoid.

### Reconstitution with naive wild-type mouse serum or polyclonal IgM prevents the hypersusceptibility of 129S.sIgM^−/−^ mice

**FIGURE 4.**
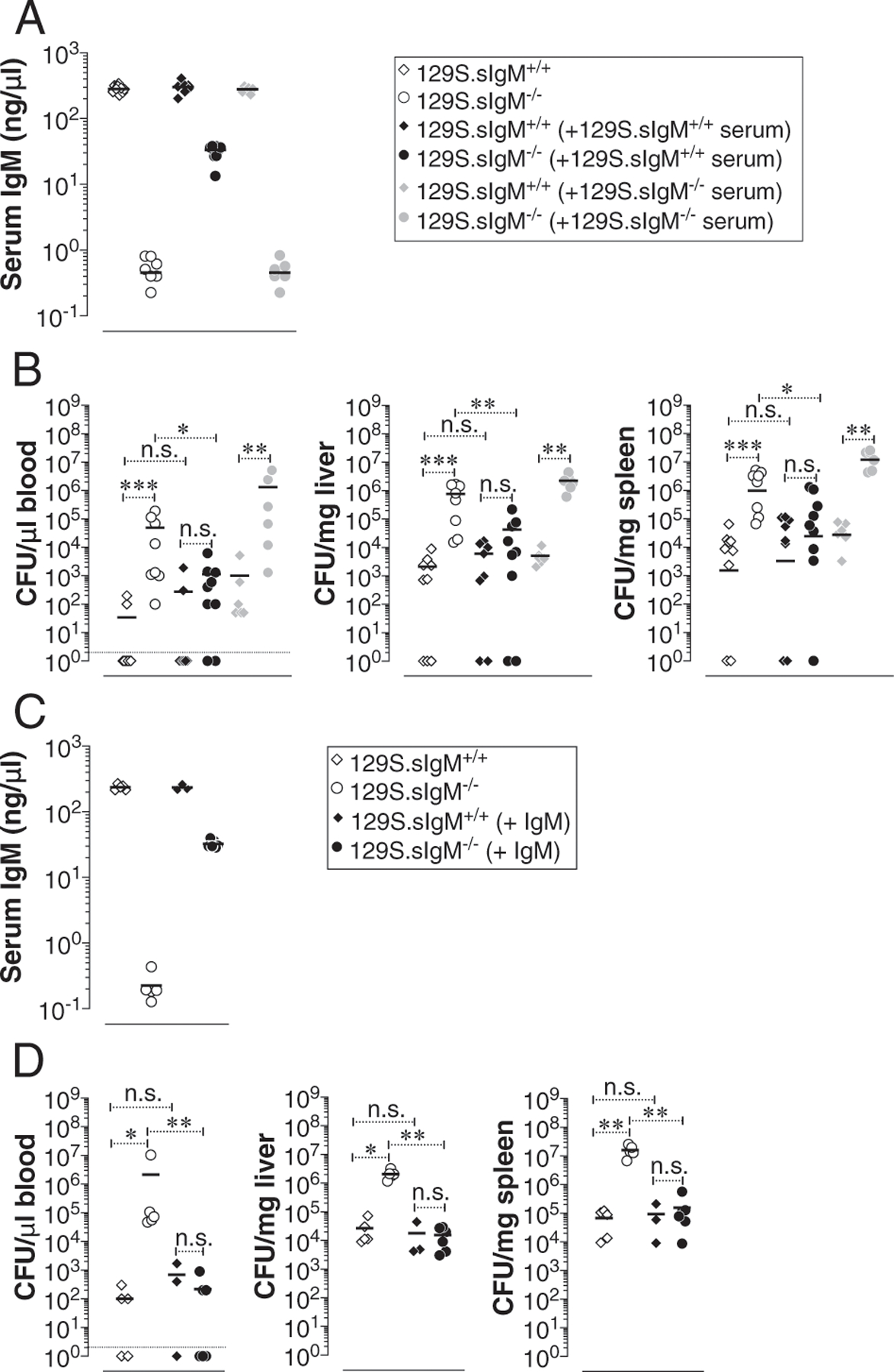
Transfer of naive wild-type serum or purified polyclonal IgM reduces the bacterial burden in mice deficient in sIgM. (**A** and **C**) 129Sv mice deficient or sufficient in sIgM were injected with 150 μl of wild-type or sIgM^−/−^ serum or 150 μg of purified polyclonal IgM i.p. and a day later total IgM levels of recipient mice were determined by ELISA. (**B** and **D**) Mice were infected i.p. with 3 × 10^4^ CFU of *S*. Typhimurium strain RC60. At 3 d postinfection, bacterial burden was determined as in Fig. 1. Each dot represents an individual mouse, and the bar represents the median. The data represent a pool of two independent experiments. Statistical differences were determined by a Mann–Whitney *U* test. *p < 0.05, **p < 0.01, ***p < 0.001. n.s., not significant.

To test whether the increased susceptibility of sIgM^−/−^ mice to *S.* Typhimurium strain RC60 infection is attributable to natural IgM deficiency, we transferred either naive wild-type or sIgM^−/−^ mouse serum, or purified IgM to 129S.sIgM^+/+^ and 129S.sIgM^−/−^ mice, prior to bacterial challenge. The serum of wild-type mice contains ~300 ng/μl IgM. By injecting 150 μl of wild-type mouse serum we restored the serum IgM levels of the 129S.sIgM^−/−^ mice to ~30 ng/μl (Fig. 4A). Remarkably, even at this level of IgM reconstitution, the susceptibility of 129S.sIgM^−/−^ mice to *S.* Typhimurium strain RC60 infection was significantly reduced as evident by a decrease in the bacterial burden in the blood, liver, and spleen (Fig. 4B). To confirm that the decreased bacterial burden in 129S.sIgM^−/−^ mice was due to natural IgM alone, we transferred 200 μg of commercially available purified IgM from normal mouse serum. Coincidentally, we found similar levels of IgM (~30 ng/μl) reconstitution when we transferred 200 μg of purified IgM (Fig. 4C) as we found with 150 μl of whole serum transfer (which contains ~45 μg of IgM) (Fig. 4A). This level of purified polyclonal IgM reconstitution was sufficient in reducing the 129S.sIgM^−/−^ mice hypersusceptibility to that of 129S.sIgM^+/+^ mice as evident by a corresponding decrease in the bacterial burden in blood, liver, and spleen (Fig. 4D). These data indicate that natural IgM in naive mouse serum plays a significant role in controlling the severity of typhoid.

**FIGURE 5.**
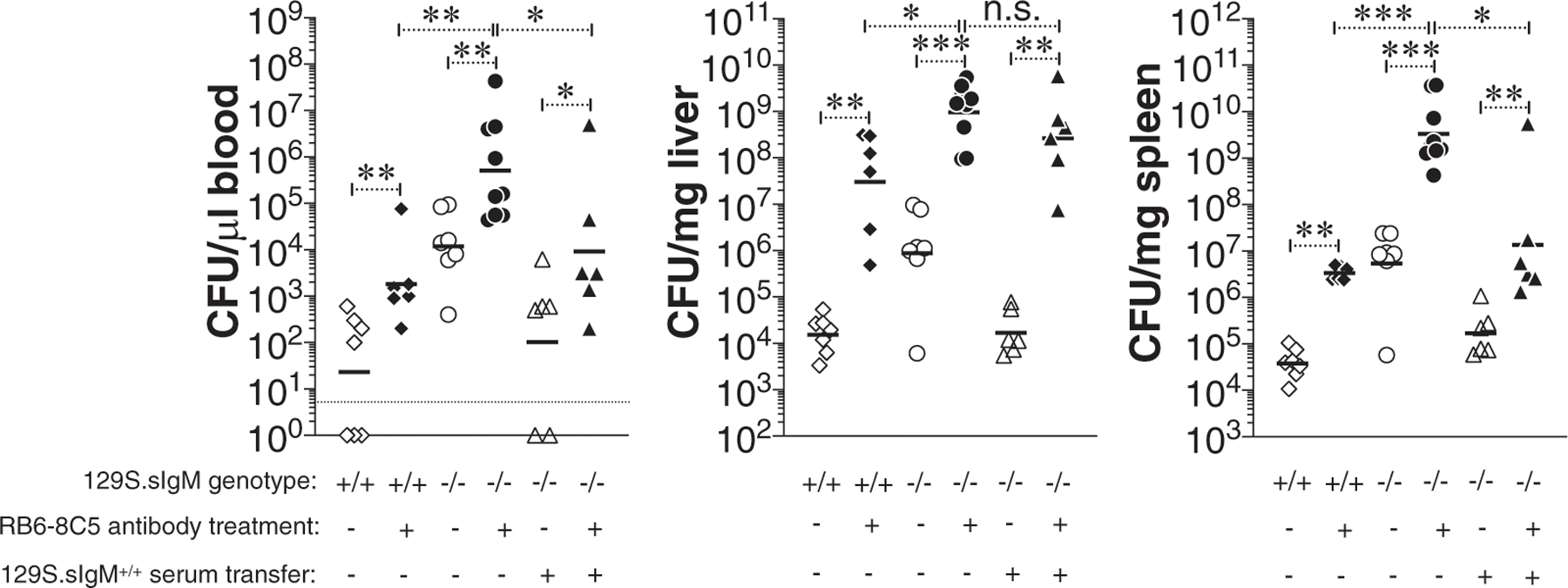
Natural IgM does not rescue the hypersusceptibility in neutrophil-depleted IgM-deficient mice. 129S.sIgM^+/+^ or 129S.sIgM^−/−^ mice were treated with anti–GR-1 mAbs i.p. to deplete neutrophils. On the same day some 129S.sIgM^−/−^ mice were also given 129S.sIgM^+/+^ serum as in Fig. 3A. A day later, all mice were infected i.p. with 3 × 10^4^ CFU of *S*. Typhimurium strain RC60. At 3 d postinfection, bacterial burden was determined as in Fig. 1. Each dot represents an individual mouse, and the bar represents the median. The data represent a pool of two independent experiments. Statistical differences were determined by a Mann–Whitney *U* test. *p < 0.05, **p < 0.01, ***p < 0.001. n.s., not significant.

Neutrophils play an important role in the protective immunity during early phases of *Salmonella* infection (58, 59). Depletion of neutrophils in mice increases the bacterial load in the blood, liver, and spleen (58, 59). FcμR (also known as TOSO), an Fc receptor for IgM, is expressed on neutrophils (60). To test the extent of protection mediated by natural IgM in the absence of neutrophils, we depleted neutrophils in mice sufficient or deficient in IgM. Depletion of neutrophils increased the bacterial burden in the livers more than in the blood and spleen of both 129S.sIgM^+/+^ and 129S.sIgM^−/−^ mice (Fig. 5). Interestingly, reconstitution of IgM (by transferring wild-type mouse serum) in 129S.sIgM^−/−^ mice significantly reduced the bacterial burden in the blood and spleen, but not in the liver. These data suggest that natural IgM contributes to protective immunity additively with that of the protection conferred by neutrophils (Fig. 5).

### IgM-deficient mice show impaired ViPS-specific IgG responses to vaccination

**FIGURE 6.**
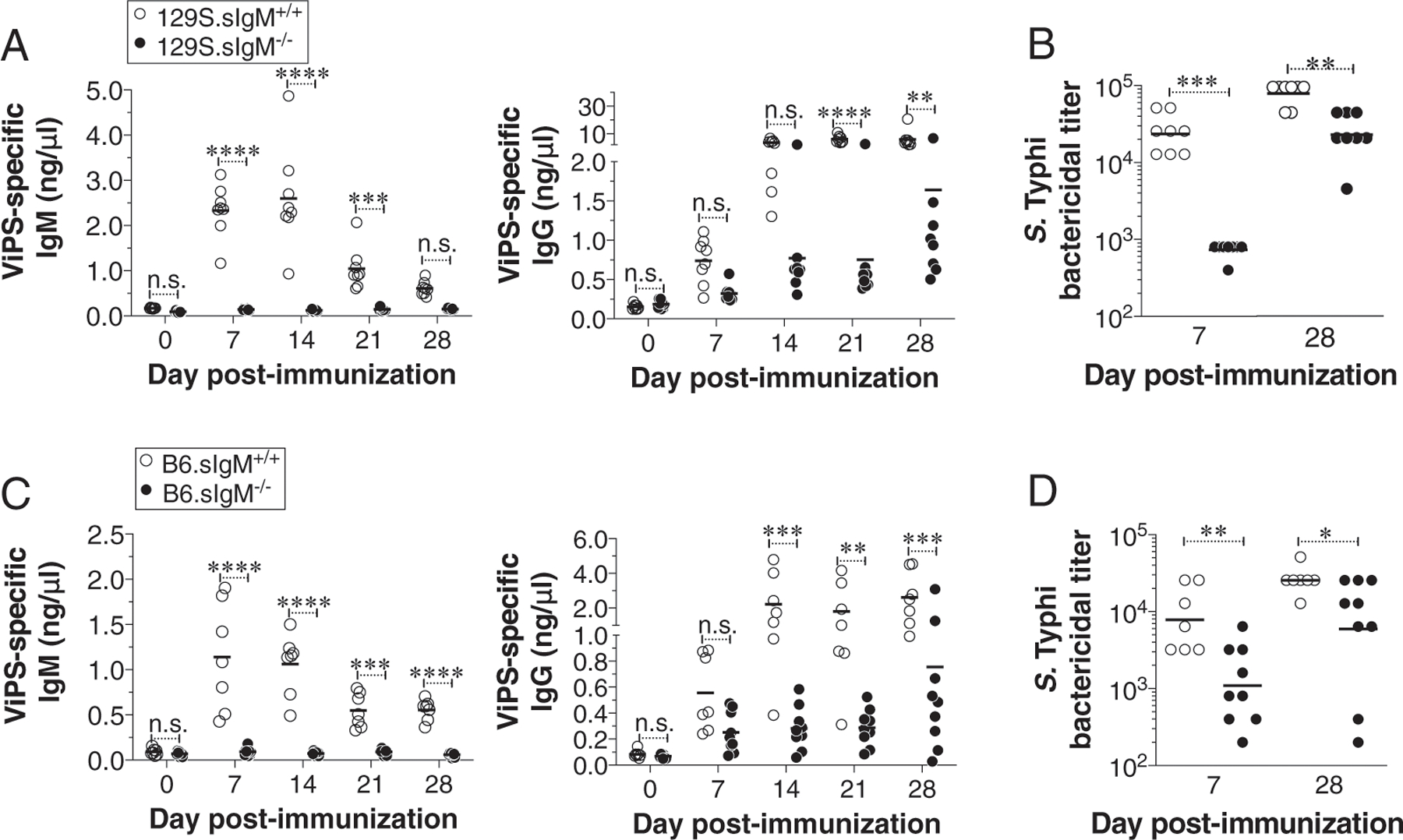
Mice deficient in sIgM generate reduced anti-ViPS and bactericidal IgG Abs. (**A** and **C**) Wild-type or mice deficient in sIgM on 129S or B6 backgrounds were immunized i.p. with heat-killed *S.* Typhi strain Ty2 (3 × 10^8^ bacterial cells), and levels of ViPS-specific IgM and IgG in the blood were measured by ELISA. Each dot represents an individual mouse, and the bar represents the mean. The data represent a pool of two independent experiments. Statistical differences were determined using a two-way ANOVA with a Bonferroni posttest. (**B** and **D**) Serum bactericidal Ab titers against *S.* Typhi strain Ty2 were determined using serum obtained from 7 and 28 d postimmunization. Each dot represents an individual mouse, and the bar represents the geometric mean. The data represent a pool of two independent experiments. Statistical differences were determined by a Mann–Whitney *U* test. *p < 0.05, **p < 0.01, ***p < 0.001, ****p < 0.0001. n.s., not significant.

sIgM^−/−^ mice express all IgG isotypes with baseline levels of IgG1, IgG2a, IgG2b, and IgG3 comparable to those of wild-type mice (42). Immunization with NP-Ficoll, a widely used synthetic polysaccharide model Ag, induces a comparable overall IgG response in wild-type and 129S.sIgM^−/−^ mice (42). However, in a lupus mouse model, the absence of sIgM causes an accelerated development of autoreactive IgG Abs (61). To test whether the lack of sIgM impacts Ab responses to ViPS, the target Ag in all typhoid subunit vaccines, we compared anti-IgG responses to ViPS using heat-killed *S.* Typhi as an immunogen. We found that mice deficient in sIgM generated a significantly lower anti-ViPS IgG compared with that in wild-type mice, regardless of the mouse strain background (Fig. 6A, 6C). To compare the functionality of the immune serum, we performed a complement-dependent serum bactericidal assay against live *S.* Typhi. We found that compared with the sIgM^+/+^ mouse serum obtained either on day 7 or day 28 postimmunization, the serum of sIgM^−/−^ mice obtained at those time points were significantly less potent in killing *S.* Typhi (Fig. 6B, 6D). These data indicate that natural IgM is also required for the generation of high titers of bactericidal anti-ViPS IgG responses.

## DISCUSSION

In mice, follicular B, MZB, B1a, and B1b cell subsets are phenotypically, developmentally, and functionally distinct (32, 37, 62, 63). A division of labor exists between B1a, MZB, and B1b cells in their ability to provide protective immunity against pathogens by producing IgM. For example, B1a and MZB cells generate most of the natural IgM that binds phosphorylcholine present on the cell wall of *Streptococcus pneumoniae*, thereby providing protection (31, 64). In contrast, B1b cells generate the Ab responses to serotype 3 polysaccharide (PPS3) of *S. pneumoniae* that can also confer protection in a serotype-specific manner (37). Human natural IgM binding to the LPS of *S*. Typhi or *S*. Typhimurium promotes respiratory burst in neutrophils in vitro (50). In the current study, we show that mouse natural IgM also binds to *S*. Typhimurium RC60 (Fig. 1) and plays an important role in protection against *S*. Typhimurium RC60 in vivo (Fig. 2). In mice, B1b cells generate most of the anti–*S.* Typhi ViPS response, which controls *S.* Typhimurium expressing ViPS in vivo (34). Our data are consistent with a nonredundant role for B1a or MZB cell–derived natural IgM and B1b-derived Ag-specific Ab responses in providing immunity against typhoid, similar to the immunity described for *S. pneumoniae* and the influenza virus (37, 65).

IgM deficiency can impact the development of the immune system in various ways in humans and mice (33, 42, 66–68). We show that reconstitution with IgM in sIgM^−/−^ mice 1 d prior to the infection prevented the hypersusceptibility (Fig. 4). Therefore, it is unlikely that an altered immune system of sIgM^−/−^ mice accounted for the hypersusceptibility to *S.* Typhimurium RC60 infection. Furthermore, the suboptimal anti-ViPS IgG responses (Fig. 6) are unlikely due to a deficiency in B1b cells, as 129S.sIgM^−/−^ and 129S.sIgM^+/+^ mice have an increased frequency of B1 cell subsets and the ratio of B1a and B1b cells is not altered (Supplemental Fig. 1) (42).

Natural IgM controls the early phases of viral and bacterial infections (69). Natural human IgM binds to LPS of *S.* Typhi and *S.* Typhimurium (50). Although IgM is an efficient isotype in activating the classical pathway of the complement system, the process requires a stable and multivalent binding on the surface of the bacterium. Using a complement-dependent bactericidal assay, we found that even at high concentrations, natural IgM does not exert any bactericidal activity (Supplemental Fig. 2). For the recruitment of C1qrs to initiate the classical pathway leading to membrane attack complex–mediated killing of the bacteria requires a stable engagement of at least two Abs with close proximity. We speculate the natural IgM binding to Ags with one of the five arms of the IgM pentamer, but the accessibility for the second arm to a nearby second epitope, might not be occurring. This explains why neither natural IgM nor natural IgG can exert any bactericidal activity. In contrast, ViPS has repetitive epitopes where Ag-specific IgM or IgG is expected to be in close proximity to recruit C1qrs and activate the classical pathway and membrane attack complex–mediated killing of the bacteria. Therefore, the mechanism of control of *S.* Typhimurium RC60 by natural IgM is independent of the classical pathway of complement activation.

Certain ViPS-conjugated vaccines do not induce optimal anti-ViPS IgG responses in typhoid disease–endemic areas (8), and it is possible that the natural IgM levels or natural IgM repertoire may be a factor to be considered in some populations. It was previously shown that natural IgM plays an important role in the initiation of Ag trapping and delivery of Ag immune complexes to the follicular dendritic cells in the spleen to initiate an optimal primary IgG Ab response (70–72). Natural IgM of humans agglutinates *S.* Typhimurium and generates immune complexes (41). In support of this, we found a robust binding of natural IgM to *S.* Typhi and *S.* Typhimurium (Fig. 1). In addition to promoting agglutination, the natural IgM may also facilitate an IgM Fc receptor–mediated opsonophagocytosis to control *S.* Typhimurium RC60 in vivo. Humans and mice express two distinct Fc receptors specific to IgM, namely Fcα/μR and FcμR (60) (73). Fcα/μR is expressed on B cells and macrophages and promote opsonophagocytosis of IgM-coated microbes (73). In contrast, FcμR is expressed on B cells and neutrophils but not macrophages (60). These cells in a Fc receptor–mediated manner may help clear Ags captured by natural IgM via opsonophagocytosis and/or transport natural IgM-complexed bacteria to the lymphoid follicles to enhance ViPS-specific IgG responses. Consistent with this, we found that the IgG responses to ViPS in the context of heat-killed *S.* Typhi immunization was significantly impaired in the absence of natural IgM (Fig. 6). Further studies are needed to understand whether these processes are mediated by natural IgM binding to its Fc receptors on neutrophils, macrophages, and/or B cells.

## Supplementary Material

Sup Fig 1 and Fig 2

## Abbreviations used in this article:

AID: activation-induced cytidine deaminase
DPBS: Dulbecco’s PBS
LB: Luria–Bertani
MZB: marginal zone B
Nramp1: natural resistance-associated macrophage protein 1
sIgM: secreted form of IgM
*S*. Typhi: *Salmonella enterica* serovar Typhi
*S.* Typhimurium: *Salmonella enterica* serovar Typhimurium
ViPS: Vi polysaccharide
