# Supplementary material for: The Lack of Natural IgM Increases Susceptibility and Impairs Anti-Vi Polysaccharide IgG Responses in a Mouse Model of Typhoid
Source: Immunohorizons. Author manuscript; Available in PMC 2023 May 23. (PMC10205010; doi:10.4049/immunohorizons.2200088)
Supplement: Sup Fig 1 and Fig 2 [file NIHMS1890180-supplement-Sup_Fig_1_and_Fig_2.pdf]

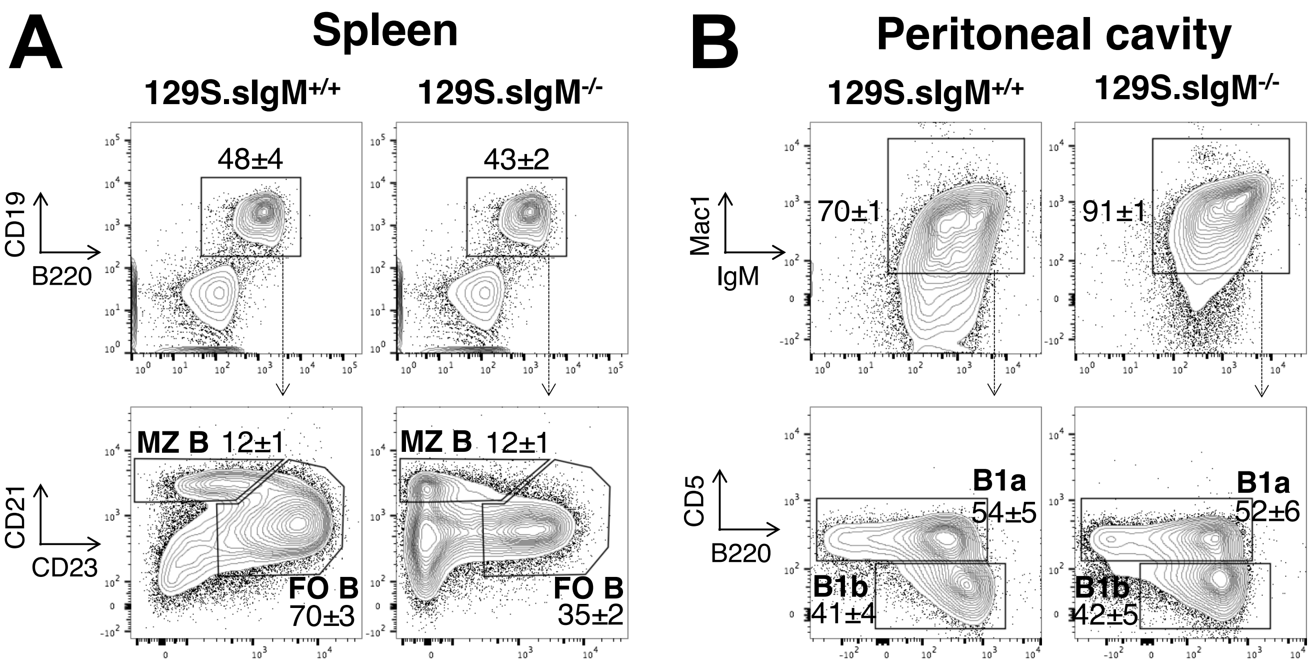

Suppl Fig 1. Flowcytometric analysis of mature B cell subsets in mice sufficient or deficient in secretory IgM. Spleen cells of mice were stained with a cocktail of antibodies specific for CD19, B220, CD21, and CD23 and analyzed by flow cytometry. Splenic B cells were first identified as $CD19^{+}$ and $B220^{+}$ cells and were resolved further as MZ ($CD23^{lo}$ and $CD21^{hi}$) and Fo ($CD23^{hi}$ and $CD21^{lo}$) B cells, and their frequencies among $CD19^{+}$ & $B220^{+}$ cells are indicated within the plots. Since B1b and B1a cells are found in abundance in the coelomic cavity of mice, peritoneal cells were stained with antibodies specific for CD19, B220, Mac1, surface IgM and CD5. All cells were first identified as B cells ($CD19^{+}$) plot not shown. B1 cells (IgM^{+} & $Mac1^{+}$ cells). The frequency of B1a ($B220^{lo/-}$ & $CD5^{+}$) and B1b ($B220^{+}$ & $CD5^{-}$) subsets were shown. All data were generated by analyzing 50,000 - 100,000 cells and are representative of 3-5 mice from each mouse strain. Five percent contour plots are shown.

Downloaded from http://journals.aai.org/immunohorizons/article-supplement/237578/pdf/immunohorizon_2200088_supplemental_1/ by Thomas Jefferson University user on 07 April 2023

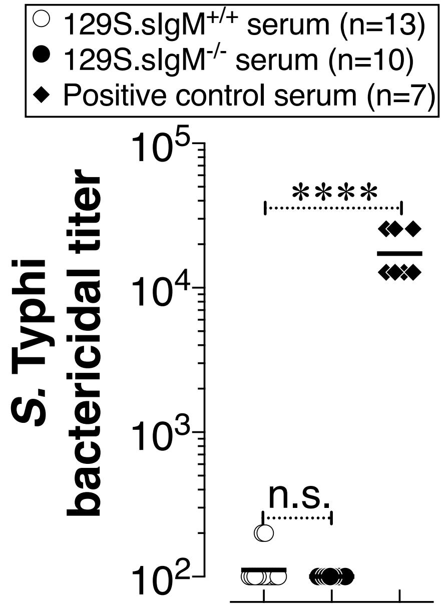

Suppl Fig 2. Natural IgM does not possess complement-dependent bactericidal activity. Serum bactericidal antibody titers against *S.* Typhi strain Ty2 were determined using the serum obtained from naïve mice sufficient or deficient in secretory IgM or serum obtained from wildtype mice immunized with heat-killed *S.* Typhi. Each dot represents an individual mouse, and the bar represents geometric mean. Statistical differences were determined by Mann-Whitney U test. Statistical differences were determined by Mann-Whitney U test. ****p<0.001, and n.s. denotes not significant.

Downloaded from http://journals.aai.org/immunohorizons/article-supplement/237578/pdf/immunohorizon_2200088_supplemental_1/ by Thomas Jefferson University user on 07 April 2023
